# Transcription Factor 21 Promotes Chicken Adipocyte Differentiation at Least in Part via Activating MAPK/JNK Signaling

**DOI:** 10.3390/genes12121971

**Published:** 2021-12-10

**Authors:** Xinyang Zhang, Bohan Cheng, Haixu Jiang, Chang Liu, Zhiping Cao, Peng Luan, Ning Wang, Hui Li

**Affiliations:** 1 Key Laboratory of Chicken Genetics and Breeding, Ministry of Agriculture and Rural Affairs, Harbin 150030, China; 18846791714@163.com (X.Z.); chengbohan1027@126.com (B.C.); S190501005@neau.edu.cn (H.J.); 13199483108@163.com (C.L.); caozhipingneau@126.com (Z.C.); luan0901@neau.edu.cn (P.L.); wangning@neau.edu.cn (N.W.); 2 Key Laboratory of Animal Genetics, Breeding and Reproduction, Education Department of Heilongjiang Province, Harbin 150030, China; 3 College of Animal Science and Technology, Northeast Agricultural University, Harbin 150030, China

**Keywords:** broiler, adipogenesis, transcription factor 21, MAPK/JNK signaling

## Abstract

The molecular mechanisms of transcription factor 21 (TCF21) in regulating chicken adipogenesis remain unclear. Thus, the current study was designed to investigate the signaling pathway mediating the effect of TCF21 on chicken adipogenesis. Immortalized chicken preadipocytes cell line (ICP), a preadipocyte cell line stably overexpressing TCF21 (LV-TCF21) and a control preadipocyte cell line (LV-control) were used in the current study. We found that the phosphorylation of c-Jun N-terminal kinases (JNK) was significantly elevated in LV-TCF21 compared to LV-control. After treating ICP cells with a JNK inhibitor SP600125, the differentiation of ICP was inhibited, as evidenced by decreased accumulation of lipid droplets and reduced expression of peroxisome proliferator-activated receptor γ (*PPARγ*), CCAAT/enhancer binding protein α (*C/EBPα*), adipocyte fatty acid binding protein (*A-FABP*)*,* and lipoprotein lipase (*LPL*). Moreover, we found that the inhibition of JNK by SP600125 remarkably impaired the ability of TCF21 to drive adipogenesis. Taken together, our results suggest that TCF21 promotes the differentiation of adipocytes at least in part via activating MAPK/JNK pathway.

## 1. Introduction

In the poultry industry, excessive fat deposition in broiler chicken is not wanted by most customers, because many metabolic diseases like coronary heart disease and arteriosclerosis are strongly related to increased dietary intake of cholesterol [1]. Increased dietary cholesterol intake from fat may lead to increased serum cholesterol levels and further increased risks of metabolic diseases [2,3]. Additionally, excessive fat deposition hinders processing and leads to significant reductions in feed efficiency and carcass yield, thus incurring economic losses for poultry producers and processors [4,5]. In addition, excessive fat deposition in chicken has increased the incidence of metabolic disorders, such as pulmonary hypertension syndrome, sudden death, fatty liver disease [6,7,8], and reduced reproductive performance, such as less sperm concentration, more sperm with abnormal morphology, and less egg production [9].

Obesity onset is closely linked with the differentiation of adipocytes [10,11]. Therefore, to facilitate therapeutic prevention or treatment of obesity, it is of great significance to get a deeper understanding of the molecular mechanisms involved in adipogenesis. Multiple distinct transcription factors and signaling pathways serve together to regulate adipogenesis [12]. Many studies have provided detailed insights into the role of transcription factors in peroxisome proliferator activated receptor (PPAR) and CCAAT/enhancer binding protein (C/EBP) family, Wnt signaling, and TGF-β signaling in this context [13,14]. To offer new insights into the molecular mechanisms of adipogenesis, identification of novel transcription factors and pathways regulating adipogenesis is particularly vital. Recently, we have identified a novel transcription factor 21 (TCF21), which promotes chicken preadipocyte differentiation by regulating the expression of lipoprotein lipase (LPL) [15]. However, the signaling pathways, by which TCF21 influences this differentiation process, remain to be characterized.

Therefore, the present study was aimed to (1) screen the signaling pathways affected by TCF21, (2) study the role of selected signaling pathway in chicken preadipocyte differentiation, and (3) perform rescue experiment to investigate whether TCF21 regulates chicken adipogenesis via the selected signaling pathway.

## 2. Materials and Methods

### 2.1. Cell Culture and Experimental Design

In this study, we utilized ICP established by infecting primary chicken preadipocytes with the recombinant retroviruses expressing chicken telomerase reverse transcriptase and telomerase RNA [16], LV-TCF21 established by infecting ICP with the recombinant lentivirus expressing chicken TCF21, and LV-control established by infecting ICP with the control lentivirus [15]. All cells were initially grown in DMEM (Gibco, NewYork, NY, USA) containing 10% FBS (Biological Industries, Kibbutz Beit Haemek, Israel) in a standard humidified incubator. When cells were >90% confluence, they were either passaged or plated for downstream experiments at 1 × 10^5^ cells/mL. When plated cells were 50% confluence, differentiation was induced by adding fresh differentiation media (DMEM containing 10% FBS and 300 μM oleic acid).

The experimental design was as follows: firstly, LV-control and LV-TCF21 cells were induced for differentiation and the accumulations of lipid droplets at 0, 24, 48, 72, 96, and 120 h after induction were assessed by oil red O staining. The earliest time point with significant difference of lipid droplet accumulation between LV-control and LV-TCF21 was selected for the following experiment. Then, luciferase reporter assay was carried out to screen signaling pathways affected by TCF21 using LV-control and LV-TCF21 cells. Additionally, Western blotting was performed to further verify the results of luciferase reporter assay. Thereafter, different concentrations (0, 2.5, 5, and 10 μM) of JNK inhibitor SP600125 was added into ICP cells to explore the role of JNK signaling in chicken adipogenesis. Finally, a rescue experiment was performed using LV-control and LV-TCF21 cells with or without supplement of SP600125. The differentiation of different groups was evaluated by the accumulation of lipid droplets and expressions of adipogenic genes using oil red O staining and RT-qPCR, respectively.

### 2.2. Oil Red O Staining and Extraction

The accumulation of lipid droplets in adipocytes was analyzed by oil red O staining. Cells were washed thrice with PBS and fixed in 4% paraformaldehyde for 30 min. Cells were again washed by PBS and then stained with freshly diluted oil red-O (oil red-O stock solution: distilled H_2_O = 3:2) for 15 min. Cells were washed five to six times with distilled water and PBS to remove excess staining. Lipid droplets were imaged using an inverted fluorescent microscope (LEICA DMIRB, Feasterville, PA, USA). Oil red O was extracted from the cells using a 100% propan-2-ol solution and measured at 510 nm. Adjacent plate wells with identical treatment were collected, and the total protein content of the cells was used to normalize the extraction results.

### 2.3. Western Blotting

**Figure 1:**
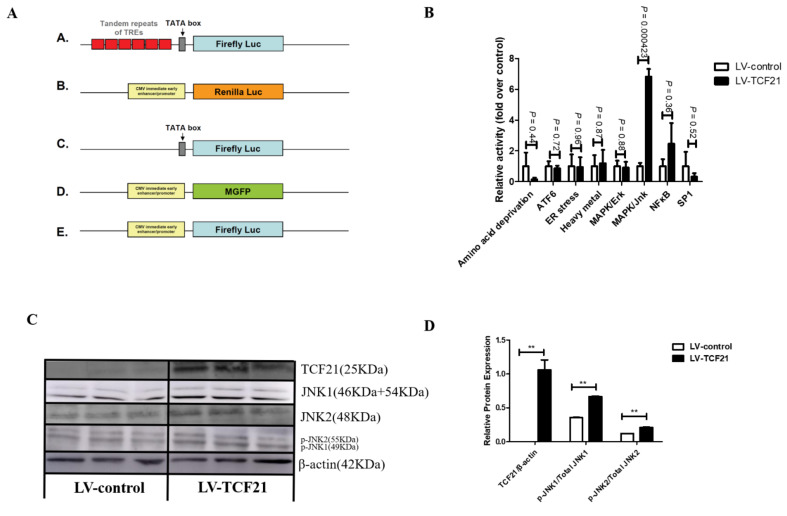
MAPK/JNK signaling pathway was activated by TCF21 overexpression. At 24 h post-induction of differentiation, lysates from LV-control and LV-TCF21 cells were collected. (**A**) A schematic overview of the constructs used for the Cignal Finder 45-Pathway Reporter Array. A. The inducible transcription factor-responsive construct expressing firefly luciferase. B. The constitutively expressing Renilla luciferase construct. C. The non-inducible firefly luciferase reporter construct. D. The constitutively expressing GFP construct. E. The constitutively expressing firefly luciferase construct. The negative control is a mixture of C. and B. (20:1). The positive control is a mixture of D., E. and B. Each reporter is a mixture of A. and B. (20:1). (**B**) A Luciferase activity-based array was used in order to identify those signaling pathways that were responsive to overexpression of TCF21. Graphs are plotted as mean ± SE relative to luciferase activity in LV-control cells from three independent experiments; (**C**) images for TCF21, JNK1, JNK2, p-JNK1, p-JNK2, and β-actin expressions in cells by Western blotting; (**D**) bands intensities were quantified by Image J software. Graphs are plotted as mean ± SE from three independent experiments. ** *p* < 0.01.

**Figure 2:**
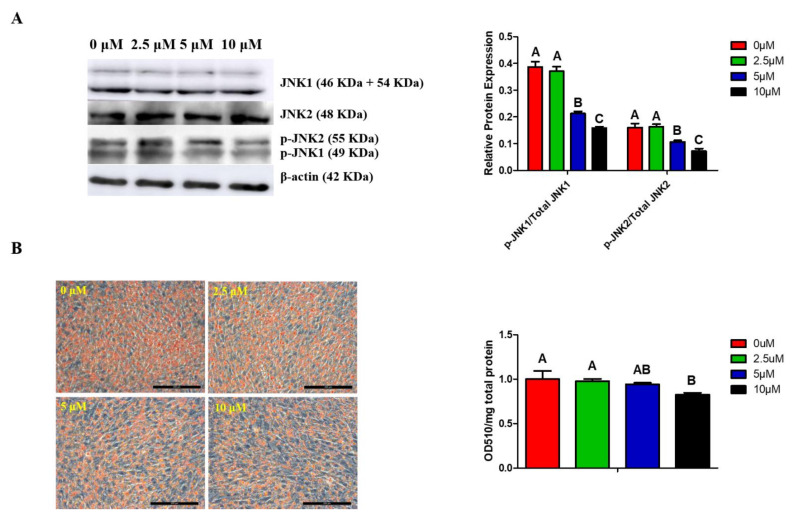
MAPK/JNK signaling and lipid droplets accumulation were inhibited by SP600125 in a dose-dependent manner. At 24 h post-induction of differentiation, ICP cells were then incubated for an additional 24 h in differentiation medium containing 0, 2.5, 5, or 10 μM SP600125. (**A**) Images for JNK1, JNK2, p-JNK1, p-JNK2, and β-actin expressions in cells treated with different concentrations of SP600126 by Western blotting (representative of three independent experiments). Then, the bands intensities were quantified by Image J software. Graphs are plotted as mean ± SE from three independent experiments. Different uppercase letters above columns denote significant differences; (**B**) images for oil-red O staining of lipid droplets in preadipocytes treated with different concentrations of SP600125 (representative of three independent experiments). Then, oil-red O dye was extracted from the cells treated with different concentrations of SP600125 in order to quantify staining intensity. Graphs are plotted as mean ± SE from three independent experiments. Different uppercase letters above columns denote significant differences.

**Figure 3:**
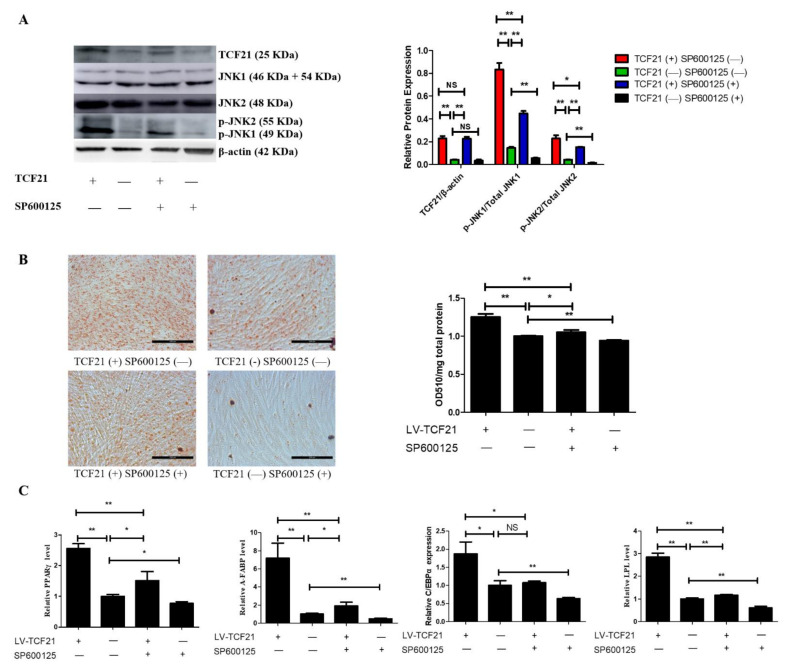
Inhibition of MAPK/JNK signaling attenuates TCF21-mediated enhancement of preadipocyte differentiation. At 24 h post-induction of differentiation, LV-TCF21 and LV-control preadipocytes were then incubated for an additional 24 h in differentiation medium containing either 0 or 10 μM SP600125. (**A**) Images for JNK1, JNK2, p-JNK1, p-JNK2, and β-actin expressions in LV-control or LV-TCF21 cells treated with 0 or 10 μM SP600126 by Western blotting (representative of three independent experiments). Then, the bands intensities were quantified by Image J software. Graphs are plotted as mean ± SE from three independent experiments. NS, no significance, * *p* < 0.05, ** *p* < 0.01; (**B**) images for oil-red O staining of lipid droplets in differentiated LV-control or LV-TCF21 preadipocytes treated with 0 or 10 μM SP600125 (representative of three independent experiments). Then, oil-red O dye was extracted from the cells in order to quantify staining intensity. Graphs are plotted as mean ± SE from three independent experiments relative to staining intensity of LV-control treated with 0 μM SP600125. * *p* < 0.05, ** *p* < 0.01; (**C**) expressions of pro-adipogenic genes in differentiated LV-control or LV-TCF21 preadipocytes treated with 0 or 10 μM SP600125 by real-time PCR. Graphs are plotted as mean ± SE from three independent experiments relative to the gene expression in LV-control treated with 0 μM SP600125. NS, no significance, * *p* < 0.05, ** *p* < 0.01.

Cells were washed thrice with PBS. Then, RIPA buffer (Santa Cruz, sc-364162, Dallas, TX, USA) supplemented with PMSF (Beyotime, ST506, Shanghai, China) and phosphatase inhibitor (Abcam, ab201119, Waltham, MA, USA) was added into cells for lysis. Then the proteins were extracted from cells. The protein concentration was determined by a BCA protein assay (Thermo Fisher, 23227, Rockford, IL, USA). Equal amounts of proteins in total cell extracts were separated by 12% SDS-PAGE and transferred to nitrocellulose membranes. Then, nitrocellulose membranes were blocked with 5% (wt/vol) BSA in TBST, followed by incubation with the following specific primary antibodies at 4 °C overnight: anti-Jnk1, 1:2500, Abcam, ab199380; anti-Jnk2, 1:5000, Abcam, ab134567; anti-P-Jnk, 1:1000, Abcam, ab4821; anti-β-actin, 1:1000, Beyotime, AA128; anti-TCF21, 1:50, Abmart, Customized. Blots were then incubated with respective HRP-conjugated secondary antibodies (donkey anti-goat IgG, 1:2500, Beyotime, A0181; goat anti-rabbit IgG, 1:5000, ZSGB-BIO, ZB-2301; goat anti-mouse IgG, 1:5000, ZSGB-BIO, ZB-2305. A BeyoECL Plus kit (Beyotime, P0018S) was then used to visualize protein bands with a chemiluminescence system (Sagecreation, Beijing, China) for Figure 1 and Supplementary Figure S1 and an ImageQuant LAS 500 system (GE, Piscataway, NJ, USA) for Figure 2 and Figure 3. The grey intensity values of bands were quantified using Image J software (NIH, Bethesda, MD, USA).

### 2.4. Luciferase Reporter Assay

The Cignal Finder Signal Transduction 45-Pathway Reporter Arrays (QIAGEN, CCA-901L, Germantown, MD, USA) (detailed information shown in Supplementary Table S1 and Figure 1A) were used based on provided protocols in the handbook to identify significantly activated or repressed signaling pathways caused by TCF21 overexpression. Dual-Luciferase Assay System (Promega, Madison, WI, USA) was used to assess luciferase activity based on provided directions, with Renilla luciferase activity used for normalization purposes. The reporter activities of signaling pathways >10× reporter activity of negative control were considered response to TCF21 overexpression and used for further analysis.

### 2.5. RT-qPCR

Cells were washed thrice with PBS. Then, 1 mL TRIzol (Invitrogen, Carlsbad, CA, USA) was added into each well to extract RNA from cells, after which nuclease-free water was used to dilute the RNA prior to visualization of rRNA and sample quality via electrophoresis on a denaturing formaldehyde agarose gel. Samples with a 28S:18S ratio of 1.8–2.1 were considered to be of sufficient quality for use in downstream experiments. A total of 1 µg of RNA from each of these samples was then used to produce cDNA with a PrimeScript™ RT reagent Kit with gDNA Eraser (Perfect Real Time) (Takara, RR047A, Beijing, China), after which qPCR reactions were performed with a QuantStudio 6 Flex System (Applied Biosystems, Foster, CA, USA). All reactions were conducted using FastStart Universal SYBR Green Master Mix (Roche, Indianapolis, IN, USA), with reactions in a 10 μL total volume containing 1 μL cDNA. Thermocycler settings were as follows: 95 °C for 10 min; 40 cycles of 95 °C for 15 s, 60 °C for 1 min. Samples were analyzed in triplicate, with TATA-box binding protein (TBP) used for normalization purposes. The 2^−^^△△CT^ method was used to quantify relative gene expression. Primer sequences are presented in Table 1.

### 2.6. Statistical Analysis

All data were obtained from three independent experiments with triplicate in each experiment and were shown as mean ± SE. Student’s t-tests were used to compare results between two groups. When more than two groups were compared, the Turkey’s HSE test was used. JMP v11.0 (SAS Institute Inc., Cary, NC, USA) was used for all analyses, and the threshold of significance was *p* < 0.05 or *p* < 0.01.

## 3. Results

### 3.1. Overexpression of TCF21 Leads to Enhanced Lipid Droplets Accumulation

To select a suitable time point for the following experiment, we compared the lipid accumulation between LV-control and LV-TCF21 during differentiation. First, we examined the overexpression effect of TCF21 in LV-TCF21 cells and the expression patterns of TCF21 in LV-control and LV-TCF21 cells. The results showed that both mRNA and protein expression levels of TCF21 in LV-TCF21 were significantly higher than those in LV-control before (0 h) and after induction of differentiation (24, 48, 72, 96, 120 h) (*p* < 0.01, Supplementary Figure S1). The expression patterns of TCF21 were similar in LV-control and LV-TCF21 cells that the expressions of TCF21 had an elevated trend after differentiation induction (Supplementary Figure S1). Specifically, the mRNA and protein expression patterns of TCF21 in LV-control cells were gradually increased after induction (Supplementary Figure S1). The mRNA expression pattern of TCF21 in LV-TCF21 cells was also gradually increased after induction, but its protein expression in LV-TCF21 cells had a minor decrease at 72 h after induction (Supplementary Figure S1). Meanwhile, we compared the lipid accumulation between LV-TCF21 and LV-control during differentiation in order to select an appropriate time point for the following experiment. The results showed that LV-TCF21 had remarkably more lipid droplets accumulation since 24 h post-induction of differentiation (*p* < 0.05 or *p* < 0.01, Figure S1).

### 3.2. MAPK/JNK Signaling Pathway Was Activated by TCF21 Overexpression

Based on these initial findings, we selected 24 h to screen signaling pathways that were significantly activated or repressed in response to TCF21 overexpression. Among the 45 signaling pathways we analyzed, the activity of MAPK/JNK signaling pathway was significantly elevated by TCF21 overexpression (*p* = 0.000423, Figure 1B and Supplementary Table S2). Western blotting further confirmed that TCF21 overexpression enhanced JNK phosphorylation (*p* < 0.01, Figure 1C,D).

### 3.3. MAPK/JNK Signaling and Lipid Droplets Accumulation Are Inhibited by SP600125 in a Dose-Dependent Manner

To investigate the role of MAPK/JNK signaling in chicken adipogenesis, ICP cells were treated with JNK inhibitor SP600125 at the concentrations of 0, 2.5, 5, and 10 μM, respectively. We found that the protein level of p-JNK (Figure 2A) and accumulation of lipid droplet were reduced by SP600125 in a dose-dependent manner. Additionally, the accumulation of lipid droplets in ICP cells was remarkably decreased by SP600125 at the concentration of 10 μM (Figure 2B). Therefore, SP600125 at the concentration of 10 μM was used in the following experiment.

### 3.4. Inhibition of MAPK/JNK Signaling Attenuates TCF21-Mediated Promotion of Preadipocyte Differentiation

Finally, we performed rescue experiment using LV-control and LV-TCF21 to explore whether MAPK/JNK signaling mediated the impact of TCF21 on preadipocyte differentiation. Although LV-control was derived from ICP, it was not the same as ICP due to lentivirus infection. Therefore, we examined whether SP600125 at the concentration of 10 μM was appropriate to treat LV-control cells in the rescue experiment. We found 10 μM SP600125 was also sufficient to suppress MAPK/JNK signaling and preadipocytes differentiation in LV-control cells (TCF21 (−) SP600125 (−) group vs. TCF21 (−) SP600125 (+) group in Figure 3). The results of TCF21 (+) SP600125 (−) group compared with TCF21 (−) SP600125 (−) group in Figure 3 showed that TCF21 promoted preadipocyte differentiation, as evidenced by increased lipid droplets accumulation and expressions of pro-adipogenic genes. The results of TCF21 (+) SP600125 (−) group compared with TCF21 (+) SP600125 (+) group in Figure 3 showed that inhibition of MAPK/JNK signaling attenuated the promoting effect of overexpression TCF21 on preadipocyte differentiation. The results of TCF21 (−) SP600125 (−) group compared with TCF21 (+) SP600125 (+) group in Figure 3 showed that the inhibition of MAPK/JNK signaling by SP600125 could not completely neutralize the promoting effect of overexpression TCF21 on preadipocyte differentiation.

## 4. Discussion

In the poultry industry, excessive fat deposition remains a significant problem [17], leading to significant reductions in feed efficiency and carcass yield, thus incurring economic losses for poultry producers [4]. Therefore, a better understanding of the molecular mechanisms governing poultry adipose tissue development may be of value for poultry breeding purposes.

It has been reported that TCF21 is not expressed in brown preadipocytes and is instead specific to white preadipocytes TCF21 [18], and compared with brown adipose tissues, the expression of TCF21 is more abundant in white adipose tissues [19]. In addition, TCF21 expression is significantly higher in the visceral adipose tissue of obese Uygurs [20] and obese mice [21] compared to their normal weight counterparts. These results indicate that TCF21 plays an important role in white adipose tissue development.

We recently found that TCF21 was a novel regulator that promoted chicken preadipocyte differentiation, acting at least in part via targeting and promoting the expression of LPL [15]. However, at present, the mechanisms whereby TCF21 influences adipogenesis remain poorly understood. As such, further research is needed to understand what signaling pathways are regulated by TCF21, which proteins interact with TCF21 to modulate target gene expression and regulation of adipogenesis, and how many target genes are subject to TCF21-mediated regulation in addition to LPL. Therefore, in the present study we sought to begin to resolve these unknown issues by exploring the signaling pathways through which TCF21 promotes preadipocyte differentiation.

We first checked the expression patterns of TCF21 in LV-control and LV-TCF21 cells after differentiation induction, and found that their general trends were similar with our previous results in primary preadipocytes (Supplementary Figure S1), indicating that LV-control and LV-TCF21 cells can be used for downstream experiments. In addition, we noticed a minor difference in expression patterns of TCF21 between mRNA and protein level at 72 h after induction (Supplementary Figure S1), which may be due to post-transcriptional regulation. Thereafter, we used chicken ICP cells stably overexpressing TCF21 to reveal that TCF21 promotes preadipocyte differentiation at an early time point (24 h) after induction, and the promotive effect of TCF21 on chicken adipogenesis was observed throughout the differentiation process (24–120 h after induction) (Figure S1). These results were consistent with our previous work in primary chicken preadipocytes [15]. Thus, together with our previous results [15], we confirmed the importance of TCF21 in the process of adipogenesis in chicken.

We then used a luciferase assay system to screen signaling pathways that were affected by TCF21 overexpression at 24 h after induction. Among the 45 pathways, TCF21 overexpression significantly increased the activity of MAPK/JNK signaling (Figure 1B). We further observed that TCF21-overexpressing cells had higher levels of p-JNK (Figure 1C,D), which further confirmed the luciferase assay results in Figure 2B. In colorectal cancer [22] and cholangiocarcinoma [23], TCF21 functions as a tumor suppressor by modulating ERK and PI3K/Akt signaling. Ao et al. [24] reported that TCF21 functions as a corepressor in the ERα signaling pathway and disrupts the growth of ERα-positive breast cancer cells. Ide et al. [25] reported that TCF21 regulates kidney development by activation of the Gdnf-Ret-Wnt11 pathway, which is required for branching morphogenesis. These results, together with our findings, clearly demonstrate that TCF21 modulates multiple biological processes through different signaling pathways.

MAPK members can be divided into four conventional subgroups, including extracellular signal-regulated kinase 1/2 (ERK1/2), JNK, p38 MAPK, ERK5 and three atypical subgroups, including ERK 3/4, ERK 7/8, and nemo-like kinase (NLK) [26]. The role of conventional MAPK members in adipogenesis has been widely studied in mice [27,28,29]. Among them, JNK signaling is highly sensitive to stress conditions, and JNKs are activated in obesity in numerous metabolically important cells and tissues, such as adipose tissue, macrophages, and liver [30]. To the best of our knowledge, the role of MAPK/JNK signaling in chicken adipogenesis remains unclear. In this study, we found that inhibition of MAPK/JNK signaling by 10 μM SP600125 significantly reduced lipid droplet accumulation (Figure 2), thus indicating that MAPK/JNK signaling plays an important role in chicken adipogenesis. The role of MAPK/JNK signaling in preadipocytes differentiation has been previously studied in 3T3-L1 preadipocytes, but the results of these studies have not always been consistent [31,32,33]. Lee et al. [31] and Liu et al. [32] found that JNK inhibition led to enhanced adipocytic differentiation, whereas Kusuyama et al. [33] found JNK signaling to be essential for C/EBPδ induction during the early stages of differentiation of 3T3-L1 preadipocytes. Additionally, it was reported in foam cells that MAPK/JNK signaling could phosphorylate PPARγ [34]. The phosphorylation of PPARγ modulates the transcription activity of PPARγ and further influences adipogenesis [35]. In the current study, we found that inhibition of JNK signaling by SP600125 significantly inhibited adipogenesis and reduced the expression of PPARγ. However, whether MAPK/JNK signaling can phosphorylate PPARγ and then regulate chicken adipogenesis requires further study.

Thereafter, to investigate if TCF21 regulates chicken preadipocyte differentiation via MAPK/JNK signaling, we performed rescue experiments. We found that inhibition of JNK signaling significantly decreased the promotive effect of TCF21 on chicken adipogenesis (Figure 3) suggesting TCF21 promoted chicken preadipocyte differentiation by activating JNK signaling. Principally, however, we cannot exclude that SP600125 also inhibits kinases other than JNK in vivo, especially as Bain et al. (2003) previously reported inhibition of several kinases by SP600125 in vitro [36]. Therefore, in the future, combination use of other JNK inhibitors and siRNA of JNK may better elucidate the contribution degree of JNK signaling in TCF21 mediated adipogenesis. In addition, further studies are still required to elucidate the molecular mechanism regarding how TCF21 activates MAPK/JNK signaling and how MAPK/JNK signaling regulates chicken preadipocyte differentiation.

## 5. Conclusions

In summary, we provided novel evidence that TCF21 activates MAPK/JNK signaling to promote preadipocyte differentiation in chicken. Our findings enrich our understanding of chicken adipogenesis, and thereby have the potential to benefit efforts aimed at prevention of excessive fat deposition in chicken.

## Figures and Tables

**Table 1:** RT-qPCR primer sequences.

Gene	Accession Number	Primer Sequence (5’ to 3’)	Product Length (bp)
*PPARγ*	NM_001001460	F:GTGCAATCAAAATGGAGCC	170
		R:CTTACAACCTTCACATGCAT	
*C/EBPa*	NM_001031459	F:GCGACATCTGCGAGAACG	266
		R:GTACAGCGGGTCGAGCTT	
*A-FABP*	NM_204290	F:ATGTGCGACCAGTTTGT R:TCACCATTGATGCTGATAG	143
*LPL*	NM_205282	F:ATGTTCATTGATTGGATGGAGGAG	159
		R:AAAGGTGGGACCAGCAGGAT	
*TBP*	NM_205103	F:GCGTTTTGCTGCTGTTATTATGAG	122
		R:TCCTTGCTGCCAGTCTGGAC	

PPARγ: peroxisome proliferator activated receptor γ; C/EBPα: CCAAT/enhancer binding protein α; A-FABP: adipocyte fatty acid binding protein; LPL: lipoprotein lipase; TBP: TATA-box binding protein.

## Data Availability

The data supporting the findings of this study are included in the article and the Supplementary Materials.

## Supplementary Materials

The following are available online at https://www.mdpi.com/article/10.3390/genes12121971/s1, Figure S1: Detection of TCF21 overexpression efficiency and its effect on lipid droplets accumulation, Table S1: Cignal Finder 45-Pathway Reporter Array Pathways and Logistics, Table S2: The luciferase reporter assay of 45 pathways in LV-control and LV-TCF21.

## Abbreviations

TCF21	transcription factor 21
ICP	immortalized chicken preadipocytes cell line
LV-TCF21	preadipocyte cell line stably overexpressing TCF21
LV-control	control preadipocyte cell line
JNK	c-Jun N-terminal kinases
PPARγ	peroxisome proliferator-activated receptor γ
C/EBPα	CCAAT/enhancer binding protein α
A-FABP	adipocyte fatty acid binding protein
LPL	lipoprotein lipase
TBP	TATA-box binding protein
ERK	extracellular signal-regulated kinase
NLK	nemo-like kinase
