# Supplementary material for: Transcription Factor 21 Promotes Chicken Adipocyte Differentiation at Least in Part via Activating MAPK/JNK Signaling
Source: Genes (Basel). 2021 Dec 10;12(12):1971. doi: 10.3390/genes12121971 (PMC8701358; doi:10.3390/genes12121971)
Supplement: Supplementary file 1 [file genes-12-01971-s001.zip › genes-1456015-supplementary.pdf]

**Supplementary Table S1. Cignal Finder 45-Pathway Reporter Array Pathways and Logistics**

| Well position | Pathway | Transcription factor |
|---|---|---|
| A 1-2 | Amino acid deprivation | ATF2/3/4 |
| A 3-4 | Androgen | AR |
| A 5-6 | Antioxidant response | Nrf1/ Nrf2 |
| A 7-8 | ATF6 | ATF6 |
| A 9-10 | C/EBP | C/EBP |
| A 11-12 | cAMP/PKA | CREB |
| B 1-2 | Cell cycle | E2F |
| B 3-4 | DNA damage | p53 |
| B 5-6 | EGR1 | EGR1 |
| B 7-8 | ER stress | CBF/NF-Y/YY1 |
| B 9-10 | Estrogen | ER |
| B 11-12 | GATA | GATA |
| C 1-2 | Glucocorticoid | GR |
| C 3-4 | Heat shock | HSF-1 |
| C 5-6 | Heavy metal | MTF-1 |
| C 7-8 | Hedgehog | Gli |
| C 9-10 | HNF4 | HNF4 |
| C 11-12 | Hypoxia | HIF-1α |
| D 1-2 | Interferon regulation | IRF1 |
| D 3-4 | Type 1 interferon | STAT1/STAT2 |
| D 5-6 | Interferon-r | STAT1 |
| D 7-8 | KLF4 | KLF4 |
| D 9-10 | Liver X | LXR |
| D 11-12 | MAPK/Erk | SRF/Elk-1 |
| E 1-2 | MAPK/Jnk | AP-1 |
| E 3-4 | MEF2 | MEF2 |
| E 5-6 | Myc | c-Myc |
| E 7-8 | Nanog | Nanog |
| E 9-10 | Notch | RBP-Jk |
| E 11-12 | NFκB | NFκB |
| F 1-2 | Oct4 | Oct4 |
| F 3-4 | Pax6 | Pax6 |
| F 5-6 | PI3K/Akt | FOXO |
| F 7-8 | PKC/$Ca^{+2}$ | NFAT |
| F 9-10 | PPAR | PPAR |
| F 11-12 | Progesterone | PR |
| G 1-2 | Retinoic acid | RAR |
| G 3-4 | Retinoid X | RXR |
| G 5-6 | Sox2 | Sox2 |
| G 7-8 | SP1 | SP1 |
| G 9-10 | STAT3 | STAT3 |
| G 11-12 | TGF-β | Smad2/3/4 |
| H 1-2 | Vitamin D | VDR |
| H 3-4 | Wnt | TCF/LEF |
| H 5-6 | Xenobiotic | AhR |
| H 7-9 | Negative control | |
| H 10-12 | Positive control | |

**Supplementary Table 2. The luciferase reporter assay of 45 pathways in LV-control and LV-TCF21**

| Pathway | LV-control (mean ± SE) | LV-TCF21 (mean ± SE) | *P*-value |
|---|---|---|---|
| Amino acid deprivation | 0.41 ± 0.37 | 0.062 ± 0.043 | 0.44 |
| Androgen | / | / | / |
| Antioxidant response | / | / | / |
| ATF6 | 0.015 ± 0.0048 | 0.013 ± 0.0027 | 0.72 |
| C/EBP | / | / | / |
| cAMP/PKA | / | / | / |
| Cell cycle | / | / | / |
| DNA damage | / | / | / |
| EGR1 | / | / | / |
| ER stress | 0.95 ± 0.72 | 0.90 ± 0.60 | 0.96 |
| Estrogen | / | / | / |
| GATA | / | / | / |
| Glucocorticoid | / | / | / |
| Heat shock | / | / | / |
| Heavy metal | 0.090 ± 0.065 | 0.11 ± 0.078 | 0.87 |
| Hedgehog | / | / | / |
| HNF4 | / | / | / |
| Hypoxia | / | / | / |
| Interferon regulation | / | / | / |
| Type 1 interferon | / | / | / |
| Interferon-r | / | / | / |
| KLF4 | / | / | / |
| Liver X | / | / | / |
| MAPK/Erk | 0.12 ± 0.043 | 0.11 ± 0.044 | 0.88 |
| MAPK/Jnk | 0.028 ± 0.0060 | 0.19 ± 0.014 | 0.000423 |
| MEF2 | / | / | / |
| Myc | / | / | / |
| Nanog | / | / | / |
| Notch | / | / | / |
| NFκB | 0.081 ± 0.037 | 0.20 ± 0.11 | 0.36 |
| Oct4 | / | / | / |
| Pax6 | / | / | / |
| PI3K/Akt | / | / | / |
| PKC/$Ca^{+2}$ | / | / | / |
| PPAR | / | / | / |
| Progesterone | / | / | / |
| Retinoic acid | / | / | / |
| Retinoid X | / | / | / |
| Sox2 | / | / | / |
| SP1 | 0.3 ± 0.28 | 0.097 ± 0.067 | 0.52 |
| STAT3 | / | / | |
| TGF-β | / | / | |
| Vitamin D | / | / | |
| Wnt | / | / | |
| Xenobiotic | / | / | |
| Negative control | 0.00057 ± 0.000067 | 0.000625 ± 0.000062 | 0.59 |

/ means that the reporter activity of the signaling pathway is < 10 × reporter activity of negative control in at least one independent experiment.

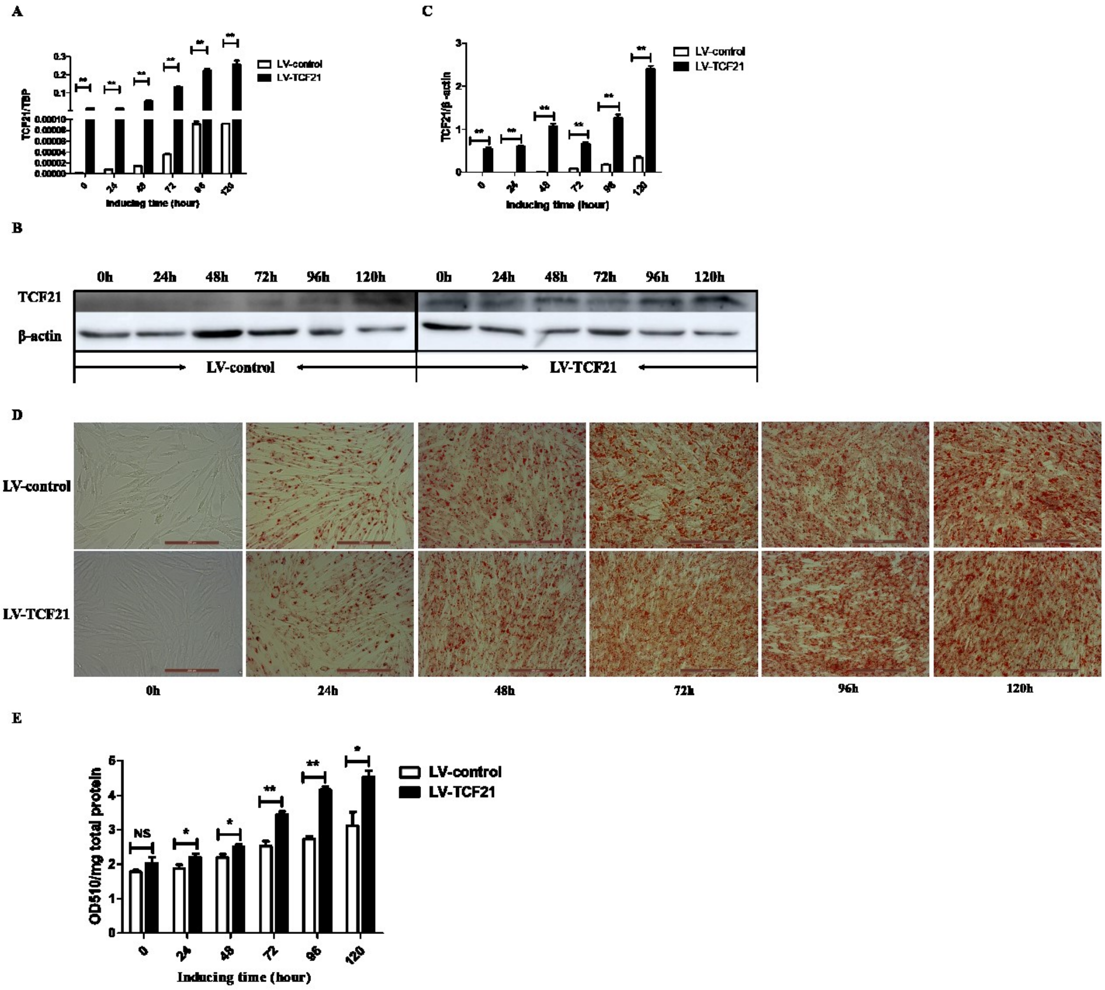


**Supplementary Figure S1. Detection of TCF21 over-expression efficiency and its effect on lipid droplets accumulation.** Oleic acid was used to induce the differentiation of LV-control and LV-TCF21 preadipocytes for 0 - 120 h. (A) The mRNA expression of TCF21 in LV-control and LV-TCF21 detected by real-time PCR. (B) Images for the protein expression of TCF21 in LV-control and LV-TCF21 detected by western blot (representative of three independent experiments). (C) The quantification of protein bands by Image J. (D) Images of the accumulation of lipid droplets in LV-control and LV-TCF21 cells by Oil red-O staining (representative of three independent experiments). (E) The Oil red-O dye was extracted from stained LV-control and LV-TCF21 preadipocytes at the indicated time points in order to quantify staining intensity. Graphs are plotted as mean ± SE from three independent experiments. NS, no significance, * $P$ <0.05, ** $P$ < 0.01.
